# Real-world progression-free survival and overall survival of palbociclib plus endocrine therapy (ET) in Japanese patients with hormone receptor-positive/human epidermal growth factor receptor 2-negative advanced breast cancer in the first-line or second-line setting: an observational study

**DOI:** 10.1007/s12282-024-01575-5

**Published:** 2024-04-20

**Authors:** Tetsuhiro Yoshinami, Shigenori E. Nagai, Masaya Hattori, Takuho Okamura, Kenichi Watanabe, Takahiro Nakayama, Hiroko Masuda, Michiko Tsuneizumi, Daisuke Takabatake, Michiko Harao, Hiroshi Yoshino, Natsuko Mori, Hiroyuki Yasojima, Chiya Oshiro, Madoka Iwase, Miki Yamaguchi, Takafumi Sangai, Nobuyoshi Kosaka, Kentaro Tajima, Norikazu Masuda

**Affiliations:** 1 Department of Breast and Endocrine Surgery, Graduate School of Medicine, Osaka University, Osaka, Japan; 2 Division of Breast Oncology, Saitama Cancer Center, Saitama, Japan; 3 Department of Breast Oncology, Aichi Cancer Center, Nagoya, Japan; 4 Department of Breast Oncology, Tokai University School of Medicine, Kanagawa, Japan; 5 Department of Breast Surgery, National Hospital Organization, Hokkaido Cancer Center, Hokkaido, Japan; 6 Department of Breast and Endocrine Surgery, Osaka International Cancer Institute, Osaka, Japan; 7 Department of Breast Surgical Oncology, School of Medicine, Showa University, Tokyo, Japan; 8 Department of Breast Surgery, Shizuoka General Hospital, Shizuoka, Japan; 9 Department of Breast Oncology, National Hospital Organization Shikoku Cancer Center, Matsuyama, Japan; 10 Department of Breast Oncology, Jichi Medical University, Shimotsuke, Japan; 11 Breast and Endocrinological Surgery, Ishikawa Prefectural Central Hospital, Kanazawa, Japan; 12 Department of Breast Surgery, Seirei Hamamatsu General Hospital, Shizuoka, Japan; 13 Department of Surgery, Breast Oncology, National Hospital Organization Osaka National Hospital, Osaka, Japan; 14 Department of Breast Surgery, Kaizuka City Hospital, Kaizuka, Japan; 15 Department of Breast and Endocrine Surgery, Nagoya University Hosipital, Nagoya, Japan; 16 Department of Breast Surgery, JCHO Kurume General Hospital, Kurume, Japan; 17 Department of Breast and Thyroid Surgery, Kitasato University School of Medicine, Kanagawa, Japan; 18 Oncology Medical Affairs, Pfizer Japan Inc., Tokyo, Japan; 19 Department of Breast and Endocrine Surgery, Nagoya University Graduate School of Medicine, 65 Tsurumai-cho, Showa-Ku, Nagoya, Aichi 466-8550 Japan

**Keywords:** Advanced breast cancer, CDK4/6 inhibitors, Palbociclib, Real-world evidence

## Abstract

**Background:**

A recent large real-world study conducted in the United States reported the effectiveness of palbociclib plus aromatase inhibitor in HR+/HER2− advanced breast cancer (ABC). However, local clinical practice and available medical treatment can vary between Japan and Western countries. Thus, it is important to investigate Japanese real-world data. This observational, multicenter study (NCT05399329) reports the interim analysis of effectiveness of palbociclib plus ET as first-line or second-line treatment for HR+/HER2− ABC by estimating real-world progression-free survival (rwPFS) and overall survival (OS) in Japanese routine clinical practice.

**Methods:**

Real-world clinical outcomes and treatment patterns of palbociclib plus ET were captured using a medical record review of patients diagnosed with HR+/HER2− ABC who had received palbociclib plus ET in the first-line or second-line treatment across 20 sites in Japan. The primary endpoint was rwPFS; secondary endpoints were OS, real-world overall response rate, real-world clinical benefit rate, and chemotherapy-free survival.

**Results:**

Of the 677 eligible patients, 420 and 257 patients, respectively, had received palbociclib with ET as first-line and second-line treatments. Median rwPFS (95% confidence interval) was 24.5 months (19.9–29.4) for first-line and 14.5 months (10.2–19.0) for second-line treatment groups. Median OS was not reached in the first-line group and was 46.7 months (38.8-not estimated) for the second-line group. The 36-month OS rates for de novo metastasis, treatment-free interval (TFI) ≥ 12 months, and TFI < 12 months were 80.2% (69.1–87.7), 82.0% (70.7–89.3), and 66.0% (57.9–72.9), respectively.

**Conclusion:**

The addition of palbociclib to ET was effective for treating HR+/HER2− ABC in Japanese routine clinical practice.

**Supplementary Information:**

The online version contains supplementary material available at 10.1007/s12282-024-01575-5.

## Introduction

Despite substantial advances in therapy, advanced breast cancer (ABC) remains an incurable disease with a 5-year survival rate of ~ 25% [1]. Of the different disease subtypes, hormone receptor-positive (HR+)/human epidermal growth factor receptor 2-negative (HER2−) tumors are the most common forms of breast cancer which are potentially sensitive to endocrine therapy (ET) [2, 3]. Current guidelines recommend the cyclin-dependent kinase 4/6 (CDK4/6) inhibitor in combination with ET in patients with HR+/HER2− ABC in the first-line and second-line settings [1, 4–7].

Palbociclib is a selective CDK4/6 inhibitor that blocks the cell cycle progression from the G1 phase to the S phase and thereby, prevents cell proliferation. In Japan, palbociclib was approved in 2017 for the treatment of HR+/HER2− inoperable or recurrent breast cancer. The safety and efficacy of palbociclib were investigated in two phase 3, randomized clinical trials (RCTs), PALOMA-2 (palbociclib plus letrozole vs placebo plus letrozole as first-line therapy [8] and PALOMA-3 (palbociclib plus fulvestrant or placebo plus fulvestrant as second-line or greater lines) [9]. Palbociclib plus ET significantly improved progression-free survival (PFS) compared to placebo in both studies, however, no statistically significant differences were observed in the overall survival (OS) [9–12] In Japanese patients with HR+/HER2− ABC (*n* = 42), a phase 2, single-arm, open-label study of palbociclib in combination with letrozole as first-line treatment demonstrated that the median PFS was 35.7 months with a manageable safety profile [13], and at a median follow-up of 89.7 months, the median OS was 85.4 months [14].

The evidence generated through RCTs may have limited generalizability because of the stringent inclusion and exclusion criteria and the limited diversity in both clinical and demographic characteristics of patients enrolled. A recent large United States (US)-based real-world study, the P-Reality X study, of OS with palbociclib plus aromatase inhibitor (AI) in HR+/HER2− metastatic breast cancer demonstrated a longer OS and PFS compared with AI alone [15]. However, local clinical practice and available medical treatment can vary between Japan and Western countries [16]; real-world evidence of palbociclib effectiveness is needed in Japan, which will provide valuable insight towards making treatment decisions. Recent reports showed the effectiveness of palbociclib among breast cancer patients in routine clinical practice in Japan and other countries as well [17–19]. However, those studies have limited number of study sites, patients, study variables, and study lines.

The present study reports the interim analysis of real-world effectiveness of palbociclib plus ET as first-line and second-line treatment of HR+/HER2− patients with ABC by estimating real-world PFS and OS in Japanese routine clinical practice.

## Patients and methods

### Study design and data source

This was a multicenter, observational study (NCT05399329) conducted in Japan. To capture the real-world clinical outcomes and treatment patterns utilizing palbociclib plus ET as first-line or second-line treatment in Japan, this study used a medical record review approach for collecting secondary data on patients with ABC from 20 sites across the country. This approach not only ensured a sufficient sample size in the limited study period but also reduced the limitations due to the different regions. This study was approved by the Institutional Review Board of Nagoya University according to the Ethical Guidelines for Medical and Health Research Involving Human Subjects issued by the Minister of Health, Labour, and Welfare (MHLW) and as per the other legal and regulatory requirements.

### Patients

All patients who initiated palbociclib plus ET in 20 study sites from December 15, 2017, to December 31, 2020, were screened. At the feasibility assessment, study sites that had experience in prescribing palbociclib for > 10 patients in both first-line and second-line settings, were chosen. Patients aged ≥ 20 years, diagnosed with HR+/HER2− ABC, and had received palbociclib plus ET in the first-line or second-line treatment were eligible. Patients with any medical records for > 6 months from palbociclib initiation were included regardless of palbociclib continuation. Also, patients with medical records for < 6 months from palbociclib initiation were included if they had records of any specific events (death, disease progression, or palbociclib discontinuation due to adverse events). This ensured that the results were not adversely affected by selective exclusion of patients with poor prognosis and/or palbociclib intolerance.

Patients were excluded if they had previously participated in or were participating in any ongoing interventional clinical trials. One induction chemotherapy (CT) regimen was allowed if the purpose of the regimen was to reduce the tumor burden, and the patients were switched to ET before disease progression. In this interim analysis, data collected from August 2022 to November 2022 were analyzed.

### Outcomes

The primary endpoint of the study was to examine the real-world PFS (rwPFS) of palbociclib plus ET. The rwPFS was defined as the time from the start of palbociclib plus ET to physician-documented disease progression or death due to any cause, whichever occurred first. The secondary endpoints included OS of palbociclib plus ET, real-world objective response rate (rwORR), real-world clinical benefit rate (rwCBR), and chemotherapy-free survival (CFS) for HR+/HER2− ABC. OS was defined as the time from the start of palbociclib treatment to death due to any cause. rwORR was defined as a documented tumor response (complete response [CR] or partial response [PR] as radiologically and/or clinically assessed by a primary physician in routine clinical practice) during treatment with palbociclib. rwCBR was defined as an achieved CR, PR, or stable disease (SD) for ≥ 24 weeks. CFS is the time from the initiation of palbociclib treatment to the start of the first subsequent CT or death due to any cause, whichever occurred first.

### Statistical methods

Patient outcomes were evaluated by treatment lines: first-line and second-line treatments. According to the Japanese Breast Cancer Society Clinical Practice Guidelines [7], the first-line treatment was defined as the first systemic therapy received for ABC regardless of recurrence timing if receiving adjuvant treatment. The subsequent treatment after the first-line treatment was defined as the second-line treatment. For reduction of tumor burden after a diagnosis of metastasis or recurrence, if a patient received induction CT and then received ET, this would still be considered a first-line treatment (first endocrine treatment). It should also be noted that induction CT in this study was only allowed if it was only one regimen and was switched to ET before disease progression. Two analysis sets were analyzed; one included all patients irrespective of starting dose of palbociclib to evaluate real-world effectiveness in clinical practice in Japan; the other included patients starting palbociclib at 125 mg/day since the starting dose in RCTs and the package insert is also 125 mg.

Subgroup analyses of rwPFS were performed, which were stratified by age, menopausal status, Eastern Cooperative Oncology Group (ECOG) score, with or without visceral metastasis, liver metastasis, bone-only metastasis, disease-free interval (DFI defined as the time from the date of breast cancer surgery to the diagnosis date of recurrence), treatment-free interval (TFI defined as the time from the end of adjuvant therapy to the diagnosis date of recurrence), and symptoms at the start of palbociclib..

Continuous variables were summarized descriptively through the tabular and graphical display of means, standard deviations, medians, and ranges of continuous variables of interest, whereas proportions and frequency distributions were used for categorical variables. The time to events, including rwPFS, OS, and CFS, were assessed by Kaplan–Meier curves and summarized in terms of medians and 95% confidence intervals (CIs). All analyses were performed using SAS version 9.4 (SAS Institute, Cary, NC, USA).

## Results

### Demographic and baseline disease characteristics of patients

**Fig. 1:**
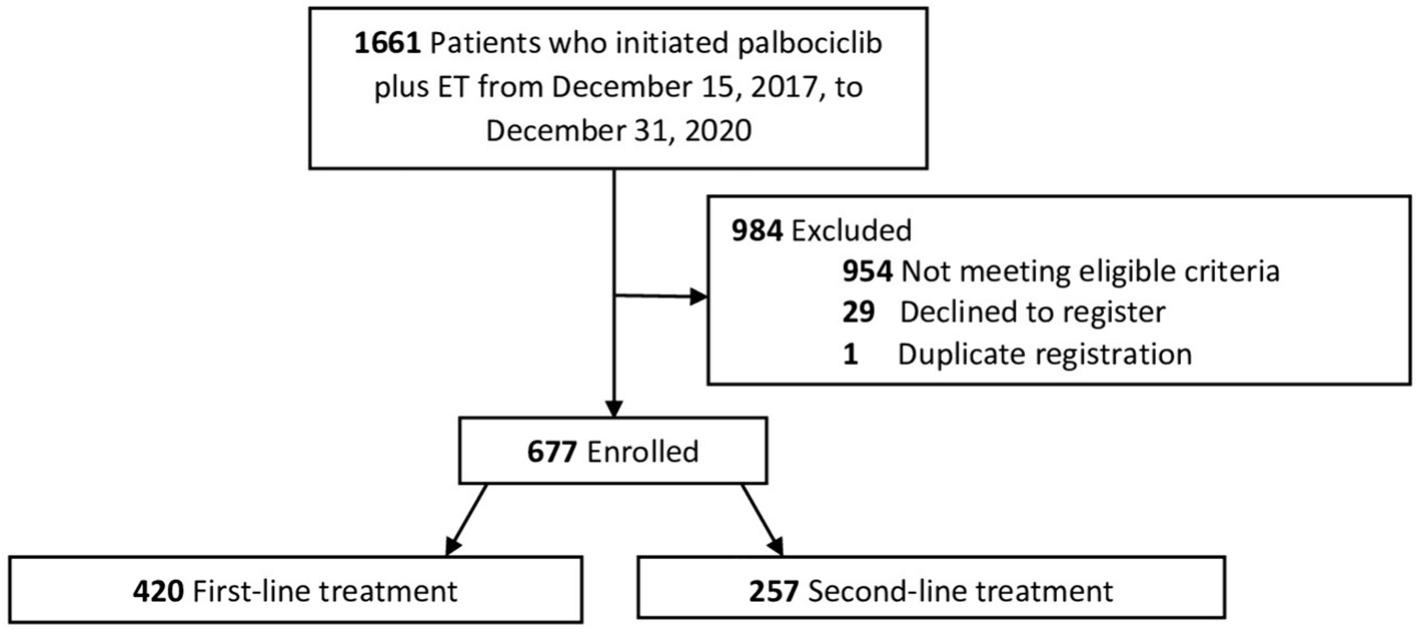
Study flow chart. ET, endocrine therapy

In total, 1661 patients who initiated palbociclib treatment between December 15, 2017, and December 31, 2020, were identified at participating sites. Of these, 677 patients met eligibility criteria; 420 patients were included in the first-line treatment group, and 257 patients were included in the second-line treatment group. Details are given in Fig. 1. The median follow-up time from palbociclib initiation was 36.3 months.

Table 1 describes the baseline characteristics of the study population. In the first-line and second-line treatment groups, respectively, the median (range) age was 60 (29–87) and 60 (32–87) years, 298 (71.5%) and 174 (68.0%) patients were postmenopausal, 23.1% and 27.2% patients exhibited stage IV disease, and 78.6% and 80.2% patients had a favorable ECOG performance status (PS) score of 0–1. Patients with low ER expression were also included; 5 patients (1.2%) in the first line setting and 4 patients (1.6%) in the second line setting.

**Table 1:** Demographic and baseline disease characteristics of patients with ABC at the start of palbociclib

Characteristics	First-line treatment, *n* = 420	Second-line treatment, *n* = 257
Age (years), median (range)	60 (29–87)	60 (32–87)
Age (years) at the start of palbociclib, *n* (%)		
20–49	86 (20.5)	56 (21.8)
50–64	178 (42.4)	98 (38.1)
65–74	116 (27.6)	64 (24.9)
≥ 75	40 (9.5)	39 (15.2)
Gender, *n* (%)		
Male	3 (0.7)	1 (0.4)
Female	417 (99.3)	256 (99.6)
Menopausal status, *n* (%)		
Pre/perimenopausal	83 (19.9)	65 (25.4)
Postmenopausal	298 (71.5)	174 (68.0)
Unknown	36 (8.6)	17 (6.6)
Stage at the time of initial treatment, *n* (%)		
0	2 (0.5)	2 (0.8)
I	56 (13.3)	32 (12.5)
II	189 (45.0)	99 (38.5)
III	68 (16.2)	42 (16.3)
IV	97 (23.1)	70 (27.2)
Unknown	8 (1.9)	12 (4.7)
ECOG PS, *n* (%)		
0	267 (63.6)	148 (57.6)
1	63 (15.0)	58 (22.6)
2	7 (1.7)	1 (0.4)
3–4	5 (1.2)	2 (0.8)
Unknown	78 (18.6)	48 (18.7)
Disease sites, *n* (%)		
Visceral metastasis	208 (49.5)	158 (61.5)
Liver metastasis	70 (16.7)	73 (28.4)
Bone-only metastasis	105 (25.0)	47 (18.3)
DFI, *n* (%)^a^		
< 24 months	39 (9.3)	23 (8.9)
≥ 24 months	275 (65.5)	157 (61.1)
TFI, *n* (%)		
De novo metastasis/others^b^	109 (26.0)	73 (28.4)
≥ 12 months	86 (20.5)	52 (20.2)
< 12 months	193 (46.0)	103 (40.1)
Number of disease sites, *n* (%)		
1	208 (49.5)	92 (35.8)
2	104 (24.8)	79 (30.7)
3	59 (14.0)	48 (18.7)
≥ 4	43 (10.2)	35 (13.6)
Symptoms at the start of palbociclib, *n* (%)		
Yes	215 (51.2)	91 (35.4)
No	189 (45.0)	155 (60.3)
Unknown	16 (3.8)	11 (4.3)
Prior CT for (neo)adjuvant, *n* (%)	215 (51.2)	121 (47.1)
Prior ET for (neo)adjuvant, *n* (%)	288 (68.6)	168 (65.4)
Induction CT, *n* (%)	23 (5.5)	8 (3.1)

*ABC* advanced breast cancer, *CT* chemotherapy, *DFI* disease-free interval (the time from the date of breast cancer surgery to the diagnosis date of recurrence), *ECOG PS* Eastern Cooperative Oncology Group performance status, *ET* endocrine therapy, *TFI* treatment-free interval (the time from the end of adjuvant therapy to the diagnosis date of recurrence)

^a^Percentage was calculated based on patients with disease stage other than “stage IV”. The patients without the date of breast cancer surgery were excluded from this calculation

^b^“Others” included patients who had surgery but did not undergo adjuvant therapy. The patients without the date of breast cancer surgery were excluded from this calculation

Further, more patients in second-line compared to first-line treatment group had visceral metastasis and liver metastasis. However, it was reverse for bone-only metastasis. About 26.0% patients in first line and 28.4% patients in second-line treatment groups had either de novo metastatic disease or belonged to the “others” category (where “others” included patients who had surgery but did not receive adjuvant therapy). Furthermore, 20.5% patients in first-line and 20.2% patients in second-line treatment groups had TFI ≥ 12 months. Conversely, 46.0% patients in first-line and 40.1% patients in second-line treatment groups had TFI < 12 months (Table 1).

### Real-world treatment pattern and dose modification of palbociclib

In the first-line vs second-line treatment groups, 90.5% vs 87.2% patients initiated palbociclib at 125 mg. Dose was reduced in 73.6% and 69.3% patients in first-line and second-line treatment groups, respectively. After dose reduction, approximately 26% patients received palbociclib at 100 mg and 40–44%, at 75 mg. At the data cutoff, 30.0% and 18.7% patients were still receiving palbociclib as first-line and second-line treatments, respectively (Table 2).

**Table 2:** Real-world treatment pattern and dose modification of palbociclib

	First-line treatment, (*n* = 420) *n* (%)	Second-line treatment, (*n* = 257) *n* (%)
Initial dose of palbociclib (mg/day)		
125	380 (90.5)	224 (87.2)
100	32 (7.6)	27 (10.5)
75	8 (1.9)	5 (1.9)
Other	0 (0.0)	1 (0.4)
Status of palbociclib administration at data cutoff^a^		
Ongoing	126 (30.0)	48 (18.7)
Discontinued	294 (70.0)	209 (81.3)
Reason for completion/discontinuation of palbociclib^b,c^		
Disease progression	200 (68.0)	162 (77.5)
Adverse event	66 (22.4)	38 (18.2)
Other	35 (11.9)	14 (6.7)
Patients requiring dose reduction^d^		
No	111 (26.4)	79 (30.7)
Yes	309 (73.6)	178 (69.3)
100 (mg/day)	109 (26.0)	68 (26.5)
75 (mg/day)	185 (44.0)	104 (40.5)
Other (mg/day)	15 (3.6)	6 (2.3)
Endocrine therapy in combination with palbociclib^e^		
Fulvestrant	237 (56.4)	197 (76.7)
Letrozole	159 (37.9)	42 (16.3)
Anastrozole	17 (4.0)	13 (5.1)
Exemestane	2 (0.5)	3 (1.2)
Tamoxifen	7 (1.7)	3 (1.2)

^a^Data cutoff date was November 7, 2022

^b^Percentage was calculated with patients who discontinued palbociclib

^c^The different reasons for palbociclib discontinuation in the same patient were counted in the respective group

^d^Percentage was calculated with patients who underwent dose reduction

^e^The different endocrine therapies used in the same patient within the same treatment line were counted in the respective group

Palbociclib administration was discontinued in 294 (70.0%) patients in first-line and 209 (81.3%) patients in second-line treatment groups. The most frequent reasons for treatment discontinuation were disease progression and adverse events (Table 2). Most patients in both groups received palbociclib in combination with fulvestrant, letrozole, and anastrozole.

### Real-world PFS and OS

**Fig. 2:**
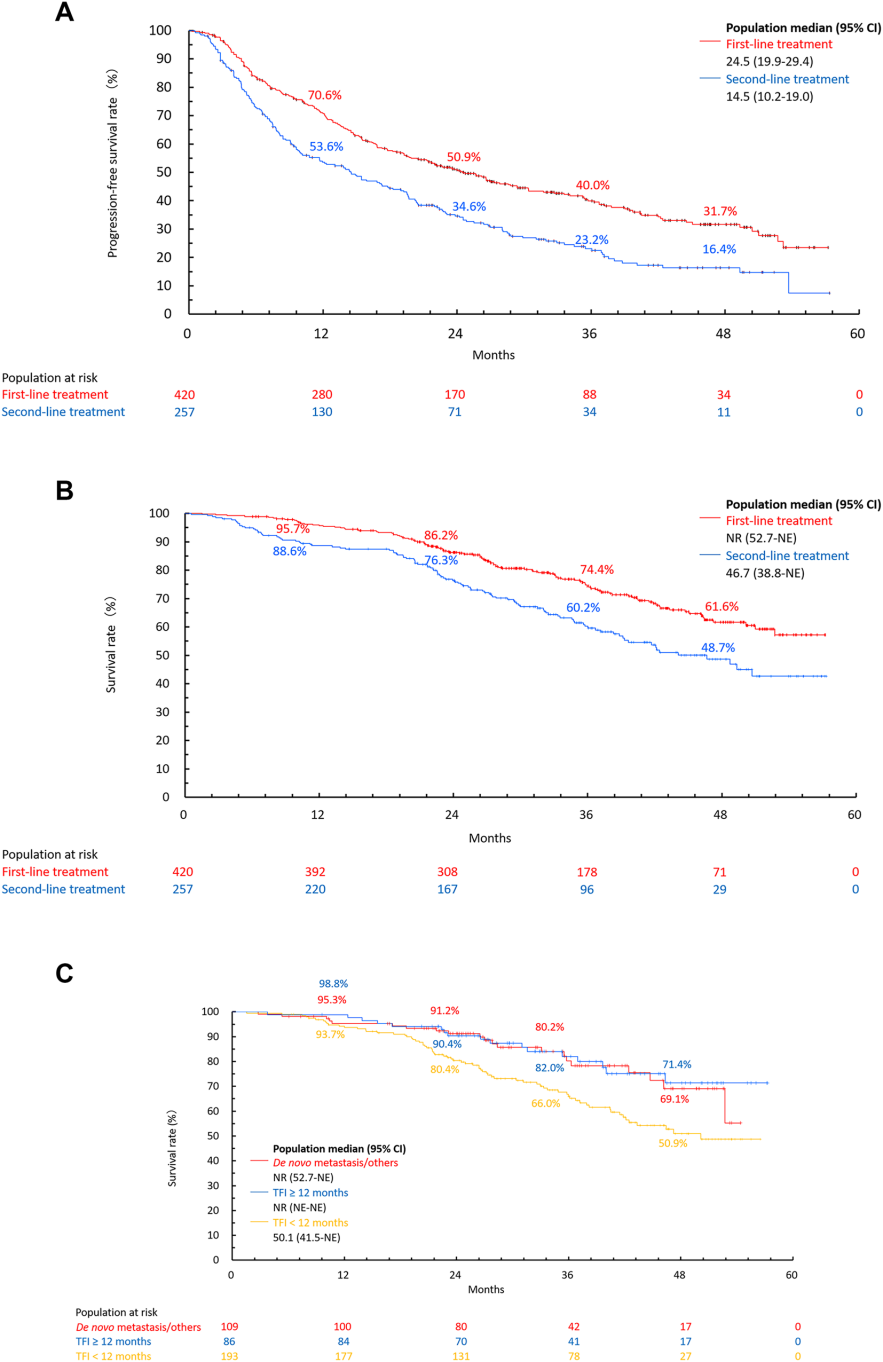
**a** rwPFS of palbociclib plus ET in first-line and second-line treatment groups. **b** OS of palbociclib plus ET in first-line and second-line treatment groups. **c** OS in patients with de novo stage VI/others^a^, TFI ≥ 12 months, and TFI < 12 months in the first-line treatment group. *CI* confidence interval, *ET* endocrine therapy, *NE* not estimated, *NR* not reached, *OS* overall survival, *rwPFS* real-world progression-free survival, *TFI* treatment-free interval (the time from the end of adjuvant therapy to the diagnosis date of recurrence). ^a^“Others” included patients who had surgery but did not undergo adjuvant therapy

Median rwPFS (95% CI) was 24.5 months (19.9–29.4) for first-line and 14.5 months (10.2–19.0) for second-line treatment groups (Fig. 2a). In subgroup analysis by baseline characteristics, median rwPFS was longer for pre/perimenopausal than postmenopausal patients (first-line: 30.4 vs 21.8 months; second-line: 16.8 vs 13.9 months). The median rwPFS was 33.6, 27.3, and 14.5 months for patients in the first-line treatment group with de novo metastatic disease, TFI ≥ 12 months, and TFI < 12 months, respectively. It was also longer for patients without liver metastasis vs those with liver metastasis. Other subgroup analyses are shown in Table 3.

**Table 3:** Subgroup analysis for summarizing median rwPFS of palbociclib plus ET in first-line and second-line treatment groups

Patient subgroup	rwPFS Median (95% CI), months	
	First-line treatment	Second-line treatment
All patients	24.5 (19.9–29.4)	14.5 (10.2–19.0)
Age (years)		
< 65	23.8 (17.8–30.4)	13.8 (9.1–19.6)
≥ 65	25.7 (19.3–35.7)	15.9 (9.9–19.7)
Menopausal status		
Pre/perimenopausal	30.4 (17.7–42.2)	16.8 (9.2–28.8)
Postmenopausal	21.8 (17.8–26.6)	13.9 (9.6–17.9)
Unknown	37.83 (7.8–51.0)	9.1 (2.8–20.6)
ECOG PS		
0	24.7 (18.9–30.4)	15.0 (11.7–19.7)
1	30.4 (17.7–NE)	13.9 (6.6–20.6)
≥ 2	16.2 (6.8–42.4)	4.5 (2.8-NE)
Unknown	24.1 (14.5–39.7)	10.2 (7.5–20.2)
Visceral metastasis		
Yes	21.3 (16.1–27.0)	13.9 (9.6–18.3)
No	27.8 (21.7–35.5)	15.4 (9.5–22.4)
Liver metastasis		
Yes	12.4 (7.9–18.9)	9.4 (7.2–13.1)
No	27.8 (22.1–35.4)	17.9 (13.8–22.8)
Bone-only metastasis	24.5 (16.7–31.7)	19.5 (9.7–24.8)
DFI (months)		
< 24	10.4 (5.6–22.7)	8.4 (5.6–20.2)
≥ 24	24.3 (18.5–30.4)	17.1 (11.7–20.5)
TFI (months)		
De novo metastasis/others^a^	33.6 (27.0–42.4)	–
≥ 12	27.3 (20.4–NE)	–
< 12	14.5 (12.1–19.2)	–
Symptoms at the start of palbociclib		
Yes	25.7 (18.2–33.6)	10.0 (6.6–17.2)
No	23.6 (17.8–29.4)	16.8 (10.8–22.2)
Unknown	38.6 (12.9–NE)	15.0 (3.7–NE)

*CI* confidence interval, *DFI* disease-free interval (the time from the date of breast cancer surgery to the diagnosis date of recurrence), *ECOG PS* Eastern Cooperative Oncology Group performance status, *NE* not estimated, *rwPFS* real-world progression-free survival, *TFI* treatment-free interval (the time from the end of adjuvant therapy to the diagnosis date of recurrence)

^a^“Others” included patients who had surgery but did not undergo adjuvant therapy

Although median OS was not reached in the first-line treatment group, median OS (95% CI) in the second-line treatment group was 46.7 months (38.8-not estimated [NE]) (Fig. 2b). Likewise, median OS (95% CI) for patients in the first-line treatment group with TFI ≥ 12 months and de novo metastasis was not reached, whereas for patients with TFI < 12 months was 50.1 months (41.5-NE) (Fig. 2c). The 36-month OS rates (95% CI) for first-line and second-line treatment groups were 74.4% (69.3–78.8) and 60.2% (53.3–66.5), respectively. The same for patients in the first-line treatment group with de novo metastasis, TFI ≥ 12 months, and TFI < 12 months were 80.2% (69.1–87.7), 82.0% (70.7–89.3), and 66.0% (57.9–72.9), respectively (Table 3 and Fig. 2c).

### rwORR, rwCBR, and CFS

**Fig. 3:**
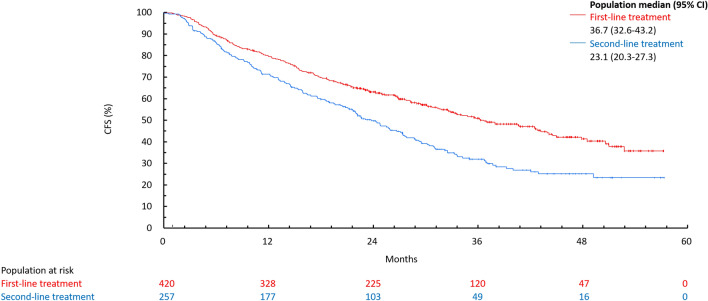
CFS of palbociclib plus ET in first-line and second-line treatment groups. *CFS* chemotherapy-free survival, *CI* confidence interval, *ET* endocrine therapy

rwORR (95% CI) was 37.9% (33.2–42.7) in first-line and 23.0% (18.0–28.6) in second-line treatment groups, whereas rwCBR (95% CI) was 76.7% (72.3–80.6) in first-line and 66.9% (60.8–72.7) in second-line treatment groups (Table 4). The median (95% CI) CFS was 36.7 months (31.8–43.9) in first-line and 23.8 months (20.5–27.4) in second-line treatment groups (Fig. 3).

**Table 4:** Summary of investigator-assessed best overall tumor response of patients with ABC in first-line and second-line treatment groups

Response	First-line treatment	Second-line treatment
CR, *n* (%)	21 (5.0)	4 (1.6)
PR, *n* (%)	138 (32.9)	55 (21.4)
SD, *n* (%)	195 (46.4)	131 (51.0)
SD ≥ 24 weeks, *n* (%)	166 (39.5)	113 (43.9)
PD, *n* (%)	49 (11.7)	55 (21.4)
rwORR (CR + PR), % (95% CI)	37.9 (33.2–42.7)	23.0 (18.0–28.6)
rwCBR (CR + PR + SD ≥ 24 weeks), % (95% CI)	77.4 (73.1–81.3)	66.9 (60.8–72.7)

*ABC* advanced breast cancer, *CI* confidence interval, *CR* clinical response, *PD* progressive disease, *PR* partial response, *rwCBR* real-world clinical benefit response, *rwORR* real-world objective response rate, *SD* stable disease

### Subgroup analysis of patients initiating palbociclib at 125 mg/day

Baseline disease characteristics of the patients who initiated palbociclib at 125 mg/day and outcomes such as rwPFS, OS, rwORR, rwCBR, and CFS are shown in Online Resources 1–8. The patient backgrounds, rwPFS, OS, and CFS were similar between overall population and patients who started treatment at 125 mg/day.

## Discussion

Real-world studies are indispensable for evaluating the effectiveness of a drug, as they allow the drug to be tested without stringent inclusion/exclusion criteria in a heterogenous population that is normally underrepresented in RCTs. Findings from real-world data (RWD) are considered mutually complementary to RCT data because RWD can provide valuable evidence by answering important questions in clinical practice that RCTs are unable to address. In this real-world study, we evaluated the effectiveness of palbociclib plus ET in Japanese patients with HR+/HER2− ABC who received the regimen as first-line or second-line therapy. Our results demonstrated the clinical benefit of palbociclib plus ET in both first-line and second-line settings, which is comparable with phase 3 studies of palbociclib [10, 15, 20]. In our understanding, this study is one of the largest RWD study of palbociclib in Japan involving 20 sites from all across Japan.

In first-line treatment group, although premenopausal patients were included in this study, the patient population in the present study was similar to those in the PALOMA-2 trial in terms of the age of patients, cancer staging, ECOG PS 0, and visceral metastases [8]. However, more patients with TFI < 12 months were enrolled in this study compared with PALOMA-2 (46.0% vs 22.3%) [8]. Accordingly, fulvestrant was the most chosen ET partner for palbociclib in Japanese clinical practice in alignment with the evidence from PALOMA-3 and Japanese breast cancer treatment guidelines [11, 21]. Similar trend was also observed in another real-world study conducted using a Japanese claim database [22]. Even though this study contained more patients with TFI < 12 months, the median rwPFS of palbociclib was consistent with PALOMA-2 (24.5 months vs 27.6 months). However, rwPFS in TFI < 12 month group (14.5 months) was shorter than that of overall study population. Furthermore, OS in the PALOMA-2 study, which was the secondary endpoint, was 53.9 months [12]. Although OS of the first-line treatment was not reached, survival rates were 74.4% at 36 months and 61.6% at 48 months.

Median OS in the first-line treatment group with TFI < 12 months was 50.1 months. Similar trend was observed in PALOMA-2 study suggesting that higher percentage of patients with TFI < 12 months led to shorter median OS in PALOMA-2 [12].

In second-line treatment group, the age of patients enrolled in the present study was similar to those enrolled in the PALOMA-3 study (median age: 57 years) [23]. Similarly, in the PALOMA-3 study, cancer staging (ECOG PS 0, 59.7%) and visceral metastases (59.4%) [24], were similar to the second-line treatment group of this study. On the other hand, in the PALOMA-3 study which included multiple treatment lines (first line 24.2%, second line 38.0%, third line 25.9%, and more than third lines 11.8%) [24], the median PFS was 11.2 months and OS was 34.8 months [9, 11]. MONARCH-2, phase 3, randomized study of abemaciclib for HR+/HER2 negative ABC patients, in which 59.4% and 38.3% of patients received treatment as first and second line, respectively [25], demonstrated a median PFS of 16.4 months and a median OS of 46.7 months [26, 27]. In this study, rwPFS was 14.5 months and OS was 46.7 months in the second-line treatment group. Taken together, these results suggested that effectiveness of palbociclib as second-line treatment was confirmed in Japanese routine clinical practice.

As shown in Table 3, patients with liver metastases had a poor prognosis despite using palbociclib as first-line in clinical practice (median PFS: with liver metastasis 12.4 months; without liver metastasis 27.8 months). Since CDK4/6 inhibitors are standard of care globally, treatment strategies specifically treating liver metastasis should be developed.

The P-Reality X study is a real-world analysis evaluating the effectiveness of first-line palbociclib plus AI vs AI alone in HR+/HER2− metastatic breast cancer in routine US clinical practice [15]. The age of patients enrolled in the present study varied from those included in the unadjusted cohort of the P-Reality X study (median age: 67 years), especially in terms of patients aged more than 65 years (this study: 37.1%, P-reality X: 61%) [15]. Likewise, in terms of cancer staging, ECOG PS 0 (37.7%), and visceral metastases (33.5%), patients in the P-Reality X study varied from those in this study. However, the rwPFS of 24.5 months in the first-line treatment group observed in our study was comparable with that observed in the P-Reality X study (19.3 months). Further, the OS rate in the first-line treatment group of 48 months (61.6%) was also similar in the P-Reality X study (52.4%). Another retrospective analysis in the US for evaluating the effectiveness and treatment patterns of first-line palbociclib plus AI reported that rwPFS and time to chemotherapy were 20 and 36.6 months, respectively [28]. This study as well as the P-Reality X study used de-identified patient data from the Flatiron Health Analytic Database, a longitudinal database that included de-identified patient data from > 280 cancer clinics [15]. On the other hand, our study used a medical record review approach for collecting secondary data on ABC patients from 20 sites across Japan. Recently, real-world studies in Asian countries have been reported, and the median rwPFS of each country is also similar [29, 30]. These results showed that palbociclib was effective even in different health care systems, different countries, and with different data collection methods.

In this study, dose reduction was performed in 73.6% patients in first-line and 69.3% in second-line treatment groups, which is comparable with the results of the Japanese subgroup population of PALOMA-2 and PALOMA3, 62.5% and 52% [31, 32]. Previous studies in patients with HR+/HER2− ABC reported that 82–86% patients initiated palbociclib at a dose of 125 mg/day in Japan [33, 34]. Moreover, the Japanese package insert of palbociclib also recommends an initial dose of 125 mg/day. In the present study, around 10% patients received doses lower than 125 mg/day as the initial dose of palbociclib, although the effectiveness of palbociclib started at 125 mg/day was similar to that in all patients included in this study (Online resources 4–8).

The rate of discontinuation of palbociclib by adverse event in this study (22.4% in first-line and 18.2% in second-line treatment groups) was higher than that in the overall population (7.4% in PALOMA-2 and 2.6% in PALOMA-3) [8, 24] or Japanese population in previous RCTs (15.6% in PALOMA-2 and 0.0% in PALOMA-3) [31, 34]. Possibly, as this study included patients who initiated palbociclib immediately after its launch in Japan, management of adverse events was not optimized in clinical practice. Another possibility is the sequential use of another CDK4/6 inhibitor, abemaciclib, which was launched in 2018 in Japan, without appropriate reduction or interruption of palbociclib. Indeed, it has been shown that abemaciclib plus fulvestrant regimen was the most commonly administered subsequent therapy after the first-line and second-line therapies (16.1% and 12.8%, respectively) in a Japanese real-world setting, demonstrated by medical claims databases in Japan [35]. In this study, of the 54 patients who received ET plus abemaciclib as the second-line treatment after first-line ET plus palbociclib, 11 switched to abemaciclib at 125 mg/day of palbociclib, and 4 switched within 2 months of starting palbociclib treatments [35], suggesting the early switching of palbociclib to abemaciclib before the dose reduction to 75 mg/day. There are several studies showing the effectiveness of sequential use of CDK4/6 inhibitors; however, the results are controversial and the evidence of sequential use of CDK4/6 inhibitors has not been solidified, waiting for the RCT for sequential use of CDK4/6 inhibitors, after the MONARCH study. Further analyses in this study are needed to prove these possibilities, such as discontinuation rate at different time points after the launch of palbociclib and the detailed analysis of palbociclib treatment pattern for patients who used abemaciclib as subsequent therapy.

As with any real-world study, limitations of our study include short duration of follow-up and lack of a control arm which lead to difficulty in interpreting results of effectiveness. Short follow-up may be able to be resolved at the timing of the final analysis, which we are planning to publish in the near future. Since this study was a medical record review, there may be missing or erroneous data entry. Moreover, disease progression and other response results were not based on standard criteria (e.g., Response Evaluation Criteria in Solid Tumors), but instead was based on the individual treating physician’s clinical assessment or interpretation of radiographic or pathologic results. Findings presented here may not be generalizable to patient populations in other countries. It should be noted that the PALOMA studies were RCTs and the data cannot be directly compared to this study.

Our findings provide evidence that the addition of the first-in-class CDK 4/6 inhibitor palbociclib to ET showed effectiveness for treating HR+/HER2− ABC in Japanese routine clinical practice.

### Supplementary Information

Below is the link to the electronic supplementary material.

Online resource 1: Demographic and baseline disease characteristics of patients with ABC who started with palbociclib 125 mg/day (DOCX 56 KB)

Online resource 2: Real-world treatment pattern and dose modification in patients with ABC who started palbociclib 125 mg/day (DOCX 53 KB)

Online resource 3: Summary of investigator-assessed best overall tumor response in patients with ABC who started palbociclib 125 mg/day (DOCX 52 KB)

Online resource 4: rwPFS of palbociclib plus ET in patients with ABC who started palbociclib 125 mg/day. ABC, advanced breast cancer; CI, confidence interval; ET, endocrine therapy; rwPFS, real-world progression-free survival (TIF 321 KB)

Online resource 5: OS of palbociclib plus ET in patients with ABC started palbociclib 125 mg/day. ABC, advanced breast cancer; CI, confidence interval; ET, endocrine therapy; NE, not estimated; NR, not reached; OS, overall survival (TIF 314 KB)

Online resource 6: CFS of palbociclib plus ET in patients with ABC who started palbociclib 125 mg/day. ABC, advanced breast cancer; CFS, chemotherapy-free survival; CI, confidence interval; ET, endocrine therapy (TIF 287 KB)

Online resource 7: rwPFS in patients with de novo stage VI/othersa, TFI ≥ 12 months, and TFI < 12 months in first-line treatment group patients who started palbociclib 125 mg/day. CI, confidence interval; NE, not estimated; rwPFS, real-world progression-free survival; TFI, treatment-free interval (the time from the end of adjuvant therapy to the diagnosis date of recurrence). ^a^“Others” included patients who had surgery but did not undergo adjuvant therapy (TIF 335 KB)

Online resource 8: OS in patients with de novo stage VI/othersa, TFI ≥ 12 months, and TFI < 12 months in first-line treatment group patients who started palbociclib 125 mg/day. CI, confidence interval; NE, not estimated; NR, not reached; OS, overall survival; TFI, treatment-free interval (the time from the end of adjuvant therapy to the diagnosis date of recurrence). ^a^“Others” included patients who had surgery but did not undergo adjuvant therapy (TIF 329 KB)

## Data Availability

Upon request, and subject to review, Pfizer will provide the data that support the findings of this study. Subject to certain criteria, conditions and exceptions, Pfizer may also provide access to the related individual de-identified participant data. See https://www.pfizer.com/science/clinical-trials/trial-data-and-results. for more information.
