# Supplementary material for: Real-world progression-free survival and overall survival of palbociclib plus endocrine therapy (ET) in Japanese patients with hormone receptor-positive/human epidermal growth factor receptor 2-negative advanced breast cancer in the first-line or second-line setting: an observational study
Source: Breast Cancer. 2024 Apr 20;31(4):621–32. doi: 10.1007/s12282-024-01575-5 (PMC11194199; doi:10.1007/s12282-024-01575-5)
Supplement: Supplementary file 1 — Online resource 1: Demographic and baseline disease characteristics of patients with ABC who started with palbociclib 125 mg/day (DOCX 56 KB) [file 12282_2024_1575_MOESM1_ESM.docx]

**Online resource 1: Demographic and baseline disease characteristics of patients with ABC who started with palbociclib 125 mg/day**

| **Characteristics** | **First-line treatment n = 380** | **Second-line treatment n = 224** |
| --- | --- | --- |
| Age (years) |  |  |
| Median, range | 59 (29-85) | 58 (31-87) |
| Gender, n (%) | | |
| Female | 378 (99.5) | 223 (99.6) |
| Male | 2 (0.5) | 1 (0.4) |
| Menopausal status, n (%) |  |  |
| Pre/perimenopausal | 82 (21.7) | 57 (25.6) |
| Postmenopausal | 262 (69.3) | 150 (67.3) |
| Unknown | 34 (9.0) | 16 (7.2) |
| Disease stage at initial diagnosis, n (%) | | |
| 0 | 1 (0.3) | 2 (0.9) |
| I | 50 (13.2) | 27 (12.1) |
| II | 172 (45.3) | 86 (38.4) |
| III | 63 (16.6) | 37 (16.5) |
| IV | 88 (23.2) | 60 (26.8) |
| Unknown | 6 (1.6) | 12 (5.4) |
| ECOG PS, n (%) | | |
| 0 | 240 (63.2) | 131 (58.5) |
| 1 | 56 (14.7) | 49 (21.9) |
| 2 | 7 (1.8) | 0 (0.0) |
| 3-4 | 5 (1.3) | 2 (0.8) |
| Unknown | 72 (18.9) | 42 (18.8) |
| Disease sites, n (%) | | |
| Visceral metastasis | 187 (49.2) | 108 (48.2) |
| Liver metastasis | 63 (33.7) | 36 (33.3) |
| Bone-only metastasis | 96 (25.3) | 60 (26.8) |
| DFI (months), n (%)^a^ | | |
| < 24 | 34 (8.9) | 5 (2.2) |
| ≥ 24 | 273 (66.1) | 152 (67.9) |
| TFI (months), n (%) | | |
| *De novo* metastasis/others^b^ | 98 (25.8) | 62 (27.7) |
| ≥ 12 | 79 (20.8) | 43 (19.2) |
| < 12 | 173 (45.5) | 91 (40.6) |

ABC, advanced breast cancer; DFI, disease-free interval (the time from the date of breast cancer surgery to the diagnosis date of recurrence); ECOG PS, Eastern Cooperative Oncology Group performance status; TFI, treatment-free interval (the time from the end of adjuvant therapy to the diagnosis date of recurrence)

^a^Percentage was calculated based on patients with disease stage other than “stage IV”.

^b^“Others” included patients who had surgery but did not undergo adjuvant therapy.
