# Supplementary material for: Real-world progression-free survival and overall survival of palbociclib plus endocrine therapy (ET) in Japanese patients with hormone receptor-positive/human epidermal growth factor receptor 2-negative advanced breast cancer in the first-line or second-line setting: an observational study
Source: Breast Cancer. 2024 Apr 20;31(4):621–32. doi: 10.1007/s12282-024-01575-5 (PMC11194199; doi:10.1007/s12282-024-01575-5)
Supplement: Supplementary file 2 — Online resource 2: Real-world treatment pattern and dose modification in patients with ABC who started palbociclib 125 mg/day (DOCX 53 KB) [file 12282_2024_1575_MOESM2_ESM.docx]

**Online resource 2: Real-world treatment pattern and dose modification in patients with ABC who started palbociclib 125 mg/day**

|  | **First-line treatment n = 380 n (%)** | **Second-line treatment n = 224 n (%)** |
| --- | --- | --- |
| Initial dose of palbociclib (mg/day) | | |
| 125 | 380 (100) | 224 (100) |
| Status of palbociclib administration at data cutoff^a^ | | |
| Ongoing | 115 (30.3) | 43 (19.2) |
| Discontinued | 265 (69.7) | 181 (80.8) |
| Reason for completion/discontinuation of palbociclib^b, c^ | | |
| Disease progression | 180 (67.9) | 140 (77.3) |
| Adverse event | 59 (22.3) | 35 (19.3) |
| Other | 32 (12.1) | 11 (6.1) |
| Patients requiring dose reduction^d^ | | |
| No | 92 (24.2) | 61 (27.2) |
| Yes | 288 (75.8) | 163 (72.8) |
| 100 (mg/day) | 109 (28.7) | 68 (30.4) |
| 75 (mg/day) | 168 (44.2) | 89 (39.7) |
| Other (mg/day) | 11 (2.9) | 6 (2.7) |
| Endocrine therapy in combination with palbociclib^e^ | | |
| Fulvestrant | 212 (55.8) | 175 (78.1) |
| Letrozole | 148 (38.9) | 37 (16.5) |
| Anastrozole | 15 (3.9) | 9 (4.0) |
| Exemestane | 2 (0.5) | 2 (0.9) |
| Tamoxifen | 5 (1.3) | 2 (0.9) |

ABC, advanced breast cancer

^a^Data cutoff date was November 7, 2022.

^b^Percentage was calculated with patients who discontinued palbociclib.

^c^The different reasons for palbociclib discontinuation in the same patient were counted in the respective group.

^d^Percentage was calculated with patients who underwent dose reduction.

^e^The different endocrine therapies used in the same patient within the same treatment line were counted in the respective group.
