# Supplementary material for: Real-world progression-free survival and overall survival of palbociclib plus endocrine therapy (ET) in Japanese patients with hormone receptor-positive/human epidermal growth factor receptor 2-negative advanced breast cancer in the first-line or second-line setting: an observational study
Source: Breast Cancer. 2024 Apr 20;31(4):621–32. doi: 10.1007/s12282-024-01575-5 (PMC11194199; doi:10.1007/s12282-024-01575-5)
Supplement: Supplementary file 3 — Online resource 3: Summary of investigator-assessed best overall tumor response in patients with ABC who started palbociclib 125 mg/day (DOCX 52 KB) [file 12282_2024_1575_MOESM3_ESM.docx]

**Online resource 3: Summary of investigator-assessed best overall tumor response in patients with ABC who started palbociclib 125 mg/day**

| **Response** | **First-line treatment** | **Second-line treatment** |
| --- | --- | --- |
| CR, n (%) | 20 (5.3) | 3 (1.3) |
| PR, n (%) | 128 (33.7) | 49 (21.9) |
| SD, n (%) | 172 (45.3) | 113 (50.4) |
| SD > 24 weeks, n (%) | 146 (38.4) | 98 (43.8) |
| PD, n (%) | 45 (11.8) | 47 (21.0) |
| rwORR (CR + PR), % (95% CI) | 39.0 (34.0-44.1) | 23.2 (17.9-29.3) |
| rwCBR (CR + PR + SD ≥ 24 weeks), % (95% CI) | 77.4 (72.8-81.5) | 67.0 (60.4-73.1) |

ABC, advanced breast cancer; CI, confidence interval; CR, clinical response; PD, progressive disease; PR, partial response; rwCBR, real-world clinical benefit response; rwORR, real-world objective response rate; SD, stable disease
