# Supplementary figures and images for: Real-world progression-free survival and overall survival of palbociclib plus endocrine therapy (ET) in Japanese patients with hormone receptor-positive/human epidermal growth factor receptor 2-negative advanced breast cancer in the first-line or second-line setting: an observational study
Source: Breast Cancer. 2024 Apr 20;31(4):621–32. doi: 10.1007/s12282-024-01575-5 (PMC11194199; doi:10.1007/s12282-024-01575-5)

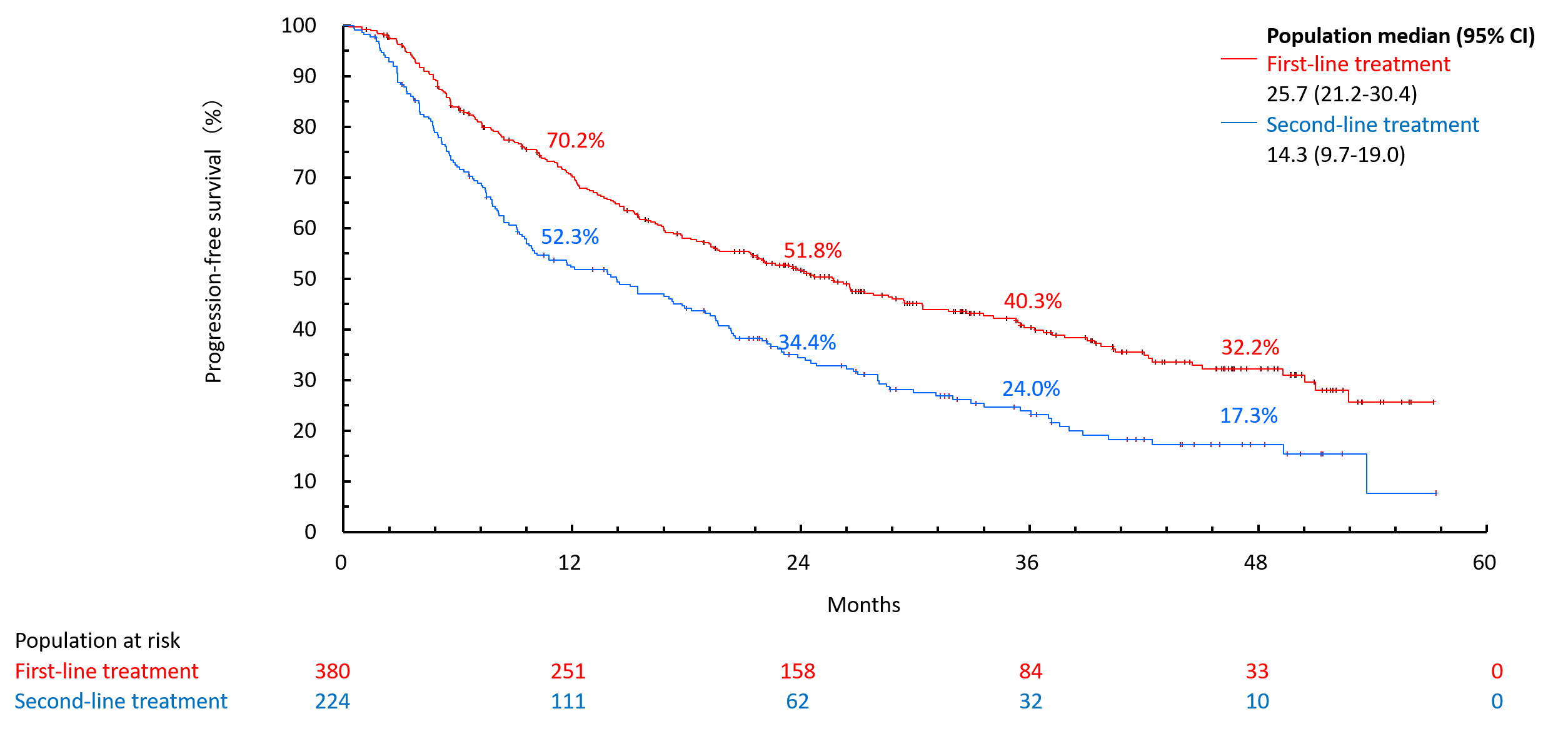

Supplement: Supplementary file 4 — Online resource 4: rwPFS of palbociclib plus ET in patients with ABC who started palbociclib 125 mg/day. ABC, advanced breast cancer; CI, confidence interval; ET, endocrine therapy; rwPFS, real-world progression-free survival (TIF 321 KB) [file 12282_2024_1575_MOESM4_ESM.tif]

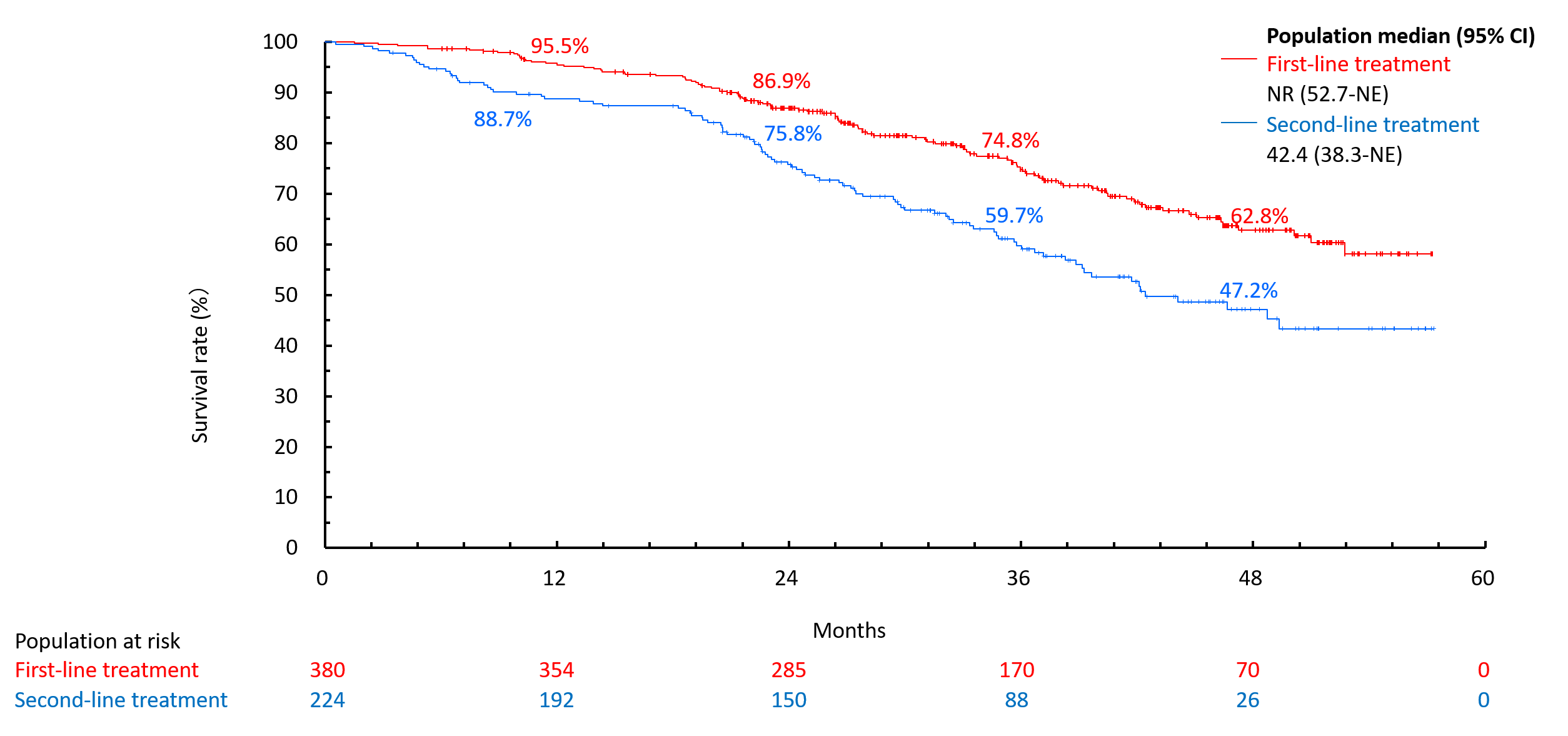

Supplement: Supplementary file 5 — Online resource 5: OS of palbociclib plus ET in patients with ABC started palbociclib 125 mg/day. ABC, advanced breast cancer; CI, confidence interval; ET, endocrine therapy; NE, not estimated; NR, not reached; OS, overall survival (TIF 314 KB) [file 12282_2024_1575_MOESM5_ESM.tif]

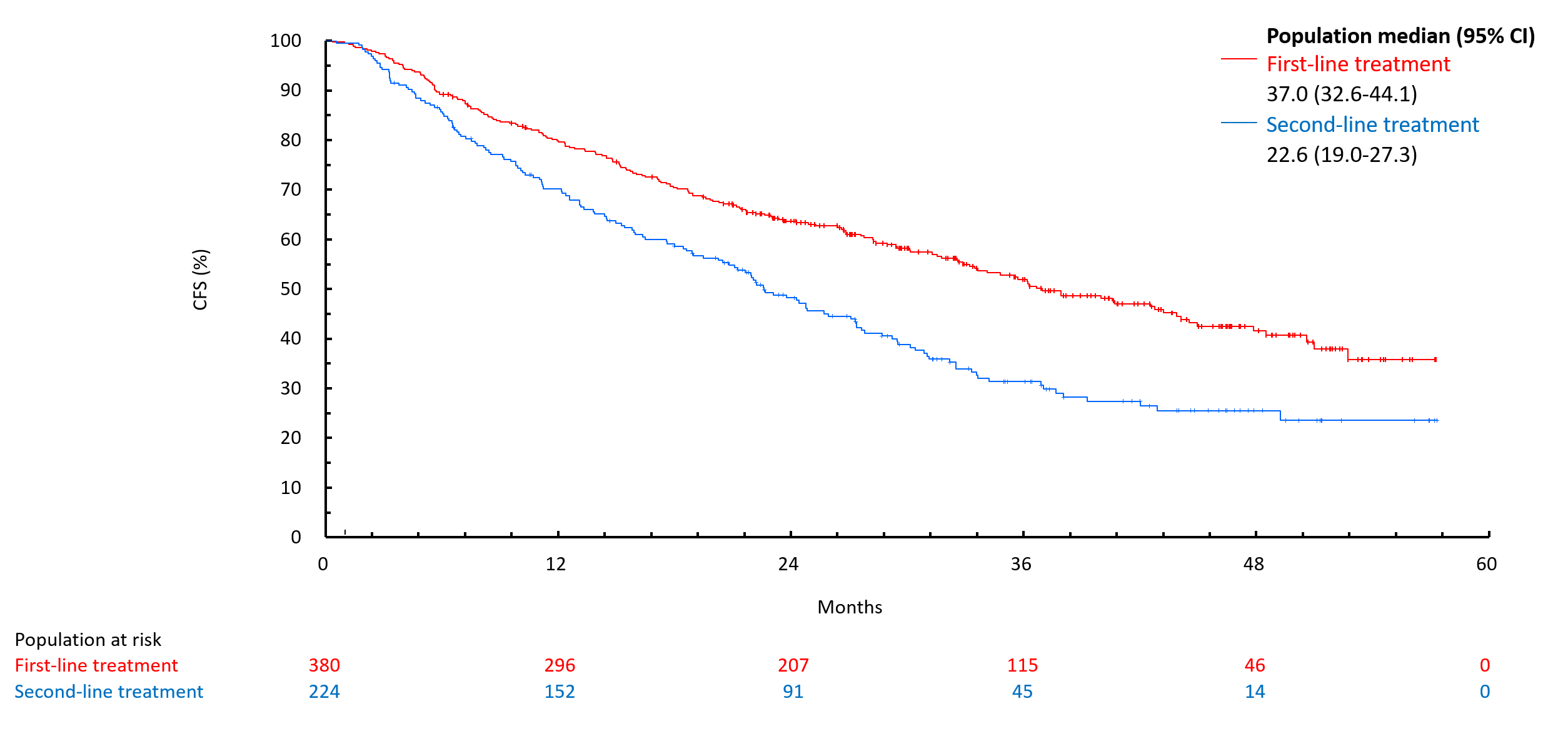

Supplement: Supplementary file 6 — Online resource 6: CFS of palbociclib plus ET in patients with ABC who started palbociclib 125 mg/day. ABC, advanced breast cancer; CFS, chemotherapy-free survival; CI, confidence interval; ET, endocrine therapy (TIF 287 KB) [file 12282_2024_1575_MOESM6_ESM.tif]

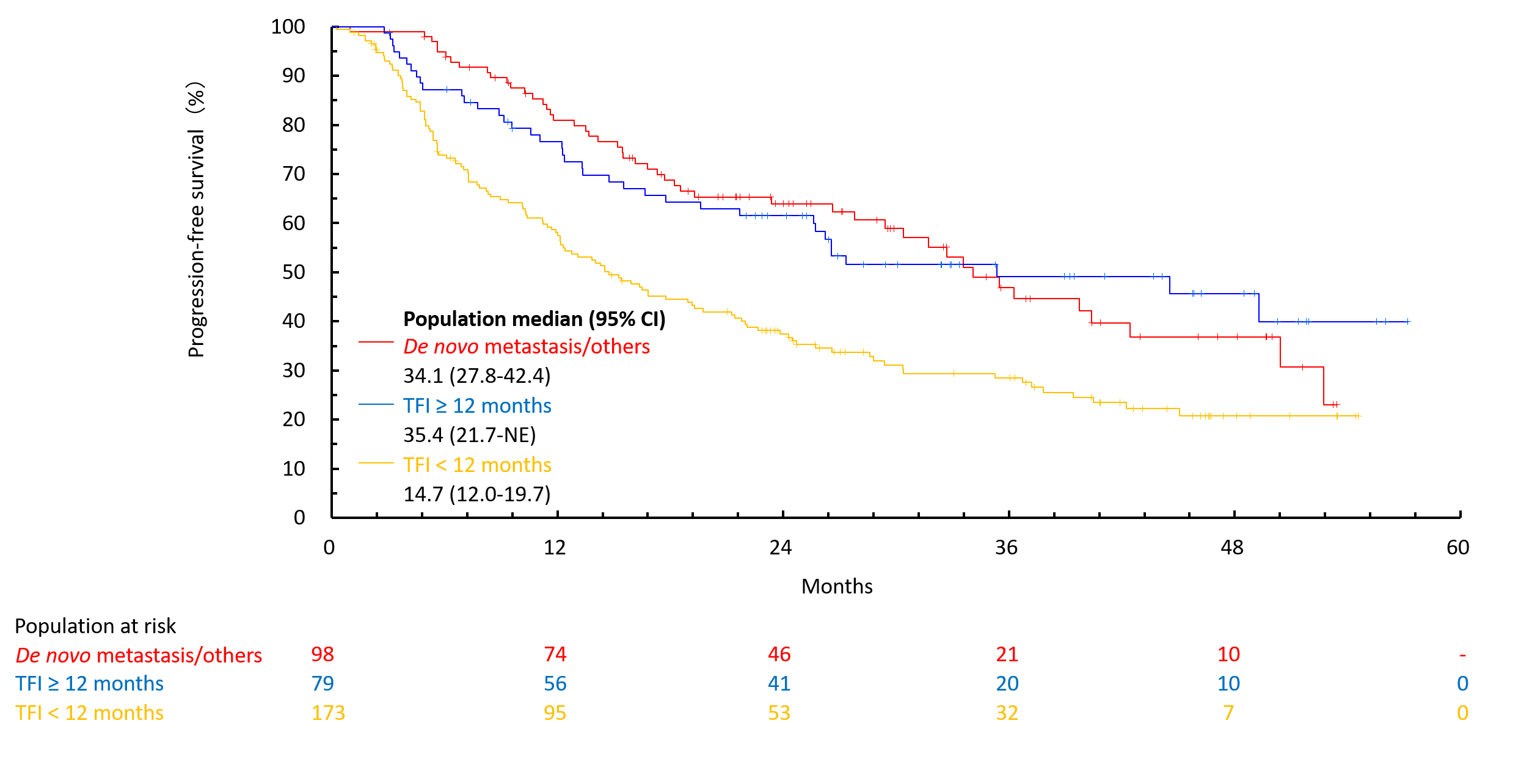

Supplement: Supplementary file 7 — Online resource 7: rwPFS in patients with de novo stage VI/othersa, TFI ≥ 12 months, and TFI < 12 months in first-line treatment group patients who started palbociclib 125 mg/day. CI, confidence interval; NE, not estimated; rwPFS, real-world progression-free survival; TFI, treatment-free interval (the time from the end of adjuvant therapy to the diagnosis date of recurrence). a“Others” included patients who had surgery but did not undergo adjuvant therapy (TIF 335 KB) [file 12282_2024_1575_MOESM7_ESM.tif]

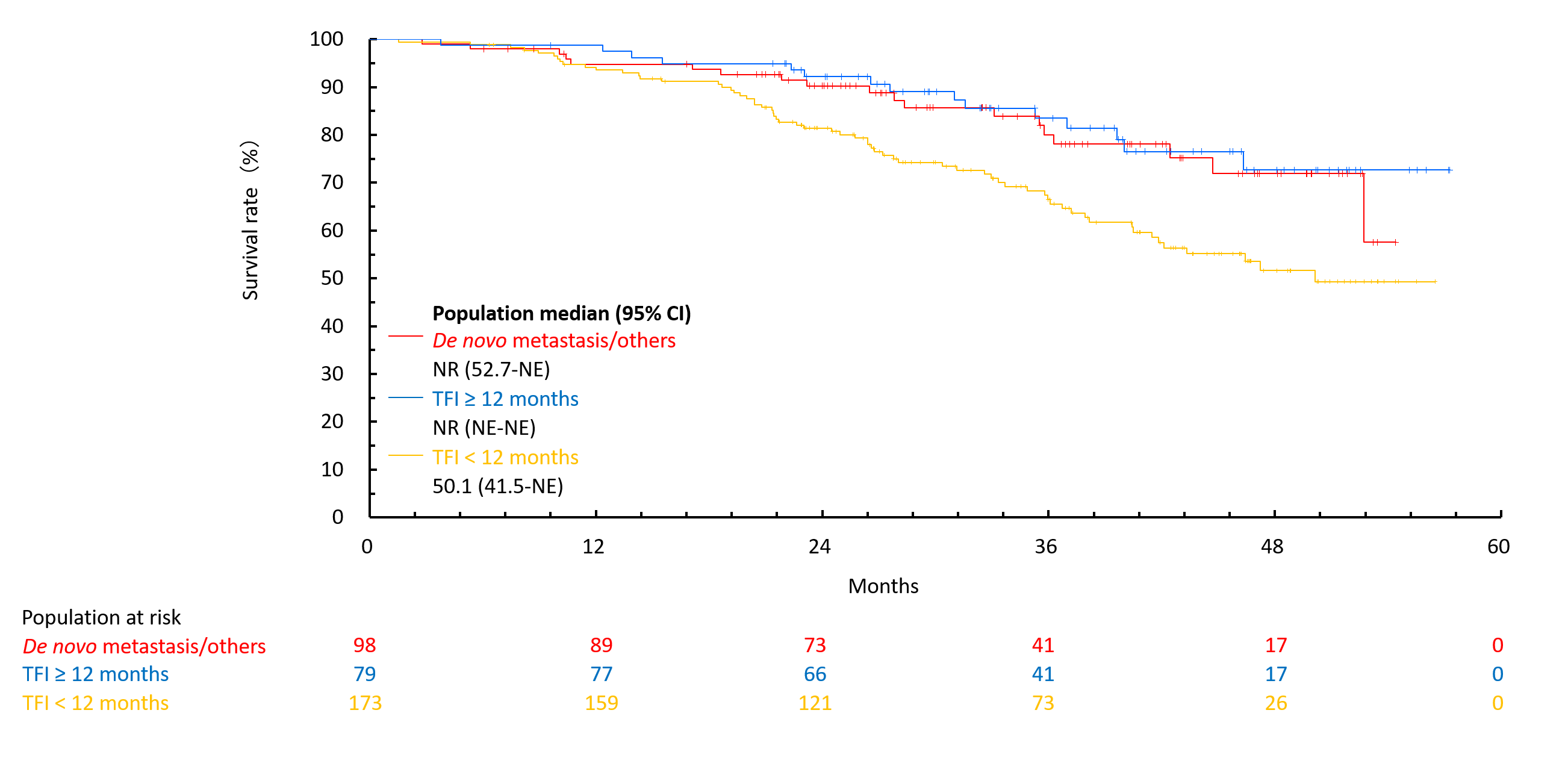

Supplement: Supplementary file 8 — Online resource 8: OS in patients with de novo stage VI/othersa, TFI ≥ 12 months, and TFI < 12 months in first-line treatment group patients who started palbociclib 125 mg/day. CI, confidence interval; NE, not estimated; NR, not reached; OS, overall survival; TFI, treatment-free interval (the time from the end of adjuvant therapy to the diagnosis date of recurrence). a“Others” included patients who had surgery but did not undergo adjuvant therapy (TIF 329 KB) [file 12282_2024_1575_MOESM8_ESM.tif]
